# Mechanochemical Preparation of Protein : hydantoin Hybrids and Their Release Properties

**DOI:** 10.1002/cssc.202102097

**Published:** 2021-12-09

**Authors:** Yusheng Yuan, Lei Wang, Andrea Porcheddu, Evelina Colacino, Niclas Solin

**Affiliations:** ^1^ Department of Physics, Chemistry, and Biology Biomolecular and Organic Electronics Linköping University 581 83 Linköping Sweden; ^2^ Department of Chemical and Geological Sciences University of Cagliari Cittadella Universitaria SS 554 bivio per Sestu 09042 Monserrato Italy; ^3^ University Montpellier CNRS, ENSCM Montpellier France

**Keywords:** drug delivery, hydantoins, mechanochemistry, protein nanofibrils, self-assembly

## Abstract

Mechanochemistry is a versatile methodology that can be employed both for covalent bond formation in organic synthesis as well as a mediator to allow preparation novel colloidal dispersions for drug delivery. Herein, ball‐milling was employed for the solid‐state preparation of fluorescent hydrophobic hydantoins, followed by the unprecedented mechanochemically‐mediated complexation of hydrophobic hydantoins within hydrophilic protein β‐lactoglobulin (BLG) and BLG nanofibrils (BLGNFs). These hydantoin:protein materials were in turn incorporated into hydrogels. The effect of incorporation of hydantoins into proteins, as well as the effect of protein structure, on the release properties were then investigated. The conversion of BLG to BLGNFs led to a more sustained release demonstrating that heat treatment of BLG into BLGNFs could be employed to modify release properties. To the best of our knowledge, this is the first example where protein : hydantoin complexes were prepared by mechanochemical methodology and mechanochemistry was combined with self‐assembly in order to prepare protein nanomaterials for drug‐delivery applications. In addition, the use of the developed protein materials is not limited to delivery of drugs but can for example be employed as components of smart food (delivery of nutrients) or release systems of pesticides.

## Graphical abstract

**Hybrid theory**: Mechanochemistry is employed to prepare fluorescently tagged hydrophobic hydantoins. These are then co‐milled with β‐lactogobulin, resulting in a water‐soluble hybrid material. By heating in acid β‐lactogobulin can be converted into nanofibrils. The state of the protein influences the release of hydantoins, with the slowest release from nanofibrils.

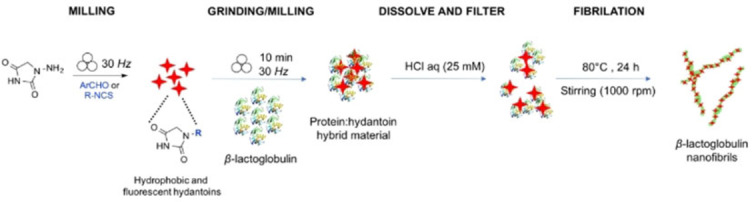

## Introduction

Over the last decades, the need for development of sustainable materials and green synthetic methodologies has encouraged researchers to design novel synthetic strategies.^[1]^ Among them, non‐conventional activation techniques such as mechanochemistry^[2]^ have received growing interest in the design of drug candidates, pharmaceutical materials, active pharmaceutical ingredients (APIs),[^3a^, ^3b^, ^3c^, ^3d^, ^3e^] organic dyes, and fluorescent molecules,[^3f^, ^3g^, ^3h^, ^3i^] opening up a new area of investigation, commonly referred to as medicinal mechanochemistry.^[4]^ An important problem of relevance in both medicine and food science is delivery of hydrophobic biologically active molecules (such as pharmaceuticals and nutraceuticals).^[5]^ Hydrophobic drugs account for over 40 % of marketed drugs and about 60 % of the therapeutic compounds at the research and development stage.^[6]^ In addition, numerous nutraceuticals (bioactives) are hydrophobic and poorly soluble in water.^[7]^ One attractive way of increasing availability of hydrophobic compounds in water is to use proteins as complexation agents.^[8]^ Common ways of achieving this are methods based on employing co‐solvents (where the hydrophobic compound is soluble) miscible with water, spray drying, or mechanical mixing (homogenization) of mixtures of protein and the hydrophobic compound in water. An interesting option is to employ mechanochemical methodology for mixing of powders of hydrophilic and hydrophobic molecules. Mechanochemical methodology is commonly employed in the pharmaceutical industry with evident advantages for formulation purposes. Indeed, the modification of the solid‐state arrangements of drugs by amorphization, or by preparing co‐crystals,^[9]^ is aimed to improve the dissolution rate, solubility, thermal and hydration stability, or compressibility of an active pharmaceutical ingredient.^[10]^ Within the context of drug delivery, mechanochemical procedures at the dry solid state present interesting advantages such as the possibility of (a) promoting the reactivity of compounds insoluble in the commonly‐used solvents and polymer carriers, and (b) preparing “homogeneous” mixtures of substances endowed with different polarities issued of hydrophilic versus hydrophobic character. For these reasons, mechanochemical procedures display a still unexplored and excellent potential for development of novel systems for delivery of hydrophobic compounds.^[11]^ When milling together hydrophobic and hydrophilic substances, the mixture is exposed to shear and/or impact forces breaking up the individual crystals resulting in metastable mixed states that can be processed further, for example, in combination with self‐assembly processes after dissolving the mixture in water. The meta‐stable states obtained after milling are then allowed to structurally develop in the aqueous phase, where the hydrophobic effect will prevent dissociation of the hydrophobic part from the soluble hydrophilic part. Such an approach, where mechanochemistry is combined with aqueous self‐assembly, has been demonstrated to be an efficient way of functionalizing both proteins^[12]^ and protein nanofibrils (PNFs)^[13]^ with various hydrophobic molecules. Milling of an excess of water‐soluble protein with the hydrophobic molecule leads to formation of a hybrid material, where the hydrophobic molecule is dispersed within the protein matrix. Upon addition of water, the protein acts as a surfactant that helps to disperse the hydrophobic material in water. If needed, the protein can be exposed to a stimulus such as heat and low pH that promotes protein self‐assembly, providing a method for tuning the properties of such protein dispersions. As the hydrophobic material is insoluble in water it remains associated with the protein, and will hence be incorporated into the self‐assembled protein structures.^[14]^ It should be noted that mechanochemical procedures involving covalent bond‐formation can also be employed; for example, by mechanochemical drug conjugation leading to self‐assembled micelles.^[11a]^ As highly complex molecules with a rich chemistry proteins are promising candidates for the development of sustainable nanomaterials, when isolated from plants (i. e., “benign by design”), industrial waste streams, or obtained as side products during food production. For example, whey‐protein is obtained as a side product during cheese production, with the main constituent being β‐lactoglobulin (BLG), a small protein built up from 162 amino acid residues with a molecular weight of about 18300 Da and an isoelectric point (pI) of 5.1. The exact biological function of BLG is still unknown, but it is believed to be involved in the transport of insoluble or chemically sensitive substances. BLG can be converted into a variety of forms by heat treatment at different pH.^[15]^ Upon heating, the protein native state becomes unstable and the protein unfolds. In the unfolded form, new bonds (e. g., hydrogen bonds or disulfide bridges) can be formed in a variety of ways. These processes occur extensively in proteins during cooking and lead to formation of new structures that will influence food texture. As a general guideline, heat treatment of BLG at a pH close to pI will result in formation of particulate gels, whereas heat treatment at a pH away from the pI will lead to formation of fibrillar objects.[^15^, ^16^] BLG can, for example, be converted into fibrils by heating aqueous solutions of BLG at a low pH (pH≈2).[^17^, ^18^, ^19^] These fibrils are structurally related to amyloid fibrils, associated with diseases such as Alzheimer's,^[19]^ even though natural protective amyloids for constructive purposes are also known, as recently reported for the so‐called functional amyloid used by some organisms.^[20]^ In addition, PNFs formed in vitro from proteins not related to diseases are generally considered as non‐pathogenic.^[21]^ Hereafter, we refer to such fibrils as protein nanofibrils (PNFs). PNFs can be formed from a wide variety of proteins, and PNFs typically have diameters of 5–10 nm and lengths of up to several micrometers,^[17]^ where the detailed structure will depend on both the specific protein and fibrillation conditions. PNFs thus constitute a type of nanomaterial and are investigated for applications in areas such as bioelectronics and biophotonics.[^22^, ^23^, ^24^, ^25^, ^26^] Furthermore, hydrogels based on PNFs were reported for biomedical applications, including drug delivery.[^27^, ^28^, ^29^] An attractive option is to develop delivery systems based on food proteins, as these often are available in large quantities at low cost and have a high nutritional value. BLG is a prominent example of a food protein whose physical properties can be changed by processes such as heating at different pH, as described above. An important class of biologically active molecules are based on the hydantoin scaffold. Hydantoins are present in a range of healthcare products (e. g., toothpaste, oral hygiene products, cosmetics, etc.) and in medicinal chemistry,^[30]^ several generic marketed drugs containing the hydantoin scaffold have been prepared in batch by mechanochemistry,[^3e^, ^3h^, ^31^] including the World Health Organization (WHO) essential medicines such as Phenytoin (antiepileptic),^[32]^ paracetamol,^[33]^ silver sulfadiazine,^[3h]^ and nitrofurantoin (antibacterial).^[3g]^ Nitrofurantoin was even prepared by continuous manufacturing at large scale by twin screw extrusion.[^34^, ^35^] Ball‐milling was also effective to prepare diverse hydantoin‐based biohybrid bridged sesquioxide nanospheres of uniform size (150 or 265 nm) by a sol‐gel mechanochemical process, outperforming the classic sol‐gel process.^[36]^

**Scheme 1:**
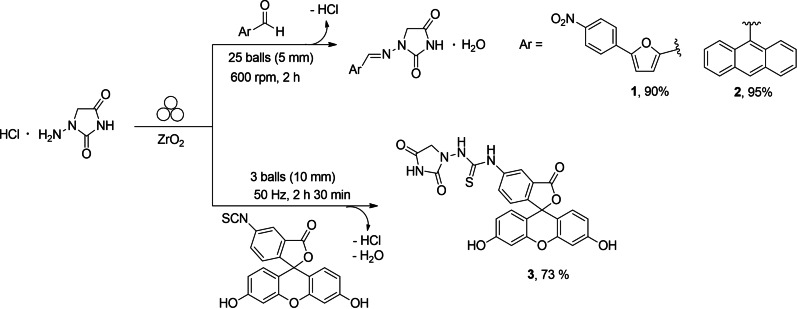
Mechanochemical preparation of hydantoin‐based dyes. For mechanochemically activated reactions, the formalism recently proposed by Rightmire and Hanusa was used.^[42]^

Herein, mechanochemical methodology is used to prepare both the hydantoin derivatives and for formulating a series of BLG protein : hydantoin hybrids to be used for drug delivery purposes. Hydantoins **1**–**3** (Scheme 1) are fluorescent compounds and can be viewed as fluorescently tagged hydantoin derivatives, selected within this study also on the basis of their different properties at different pH values. Hydantoin **1** and **2** are strongly hydrophobic, while hydantoin **3** is more polar and displays a pH‐dependent solubility. The BLG protein : hydantoin hybrids were tested for their potential as drug carrier, upon formation of complexes between (i) BLG and hydantoins **1**–**3**, respectively (hereafter labelled as **1**@BLG, **2**@BLG, **3**@BLG) and (ii) BLG PNFs (BLGNFs) and hydantoin **3** (hereafter labelled as **3**@BLGNFs). Then, after the incorporation of the BLG protein : hydantoin hybrids into a gel constituted by polyvinyl alcohol (PVA) and glycerol, the release of hydantoins **1**–**3** from the gels was monitored and the results assessed against the characteristics of the protein structure used as drug carriers (BLG vs. BLGNFs).

## Results and Discussion

### Preparation of hydantoins

Scheme 1 illustrates both the mechanochemical preparation of the myorelaxant agent dantrolene **1**, the (*E*)‐1‐((anthracen‐9‐ylmethylene)amino)imidazolidine‐2,4‐dione **2** (both previously reported by us^[3g]^), and the preparation of compound **3**.

Hydantoin derivatives **1** and **2** present a *N*‐acylhydrazone functional group, labile in strongly acidic aqueous conditions, while compound **3** is constituted by a more stable thiourea moiety. The mechanochemical synthesis of unsymmetrical thioureas was previously reported^[37]^ by a click‐type amine‐isothiocyanate coupling reaction, by employing either liquid assisted grinding (LAG)^[38]^ or dry milling conditions.^[39]^ Nevertheless, no reports deal with the mechanochemical preparation of fluorescent probes from fluoresceine isothiocyanates (FITC). A click‐type amine‐isothiocyanate coupling reaction has been employed in solution‐phase to prepare fluorescein‐tagged adamantyl^[40]^ and 3‐amino‐pyridyl thioureas,^[41]^ but the reaction scope was limited each time to one substrate. The reactions were usually conducted during 24 h under an inert atmosphere in DMF, DMSO, or THF, using an excess of triethylamine, with yields in the range of 63–70 % after column chromatography. Herein we have investigated a mechanochemical method to prepare fluorescent probes. A stoichiometric amount of FITC and 1‐amino hydantoin hydrochloride were initially ball‐milled at 30 Hz using zirconium oxide milling media (the reaction scale was 0.257 mmol). The progress of the reaction was monitored by attenuated total reflectance infrared (ATR‐IR) spectroscopy, following the disappearance of the isocyanate band at 2031 cm^−1^ (Supporting Information, Figures S1–S3). After 30 min of milling, the jar was opened and the powder analyzed in the solid‐state by ATR‐IR spectroscopy. However, even after four additional and identical consecutive milling cycles, starting material (FITC) was still present in the crude reaction mixture. The conversion could be improved by modification of reaction parameters, and full conversion could be obtained by increasing the milling frequency to 50 Hz and by employing a slight excess of 1‐amino hydantoin hydrochloride (1.2 equiv.). Under these conditions, full conversion could be obtained by continuous milling (Figures S1–S5). As for hydrazones **1** and **2**, the mechanochemical preparation of fluoresceine‐tagged aminohydantoin **3** (Scheme 1) presents several advantages compared to similar solution‐based experimental procedures:^[3g]^ (a) the use of a base to generate the more nucleophilic free amine can be avoided (reagent economy), the activation being provided by mechanochemical shocks between the reactants; (b) the reaction kinetics is improved (time economy), with full conversion of the starting materials obtained in only 150 min (instead of 24 h); (c) the experimental set‐up was simplified and no dry conditions were needed; (d) the throughput of the process and its ecological footprint were improved, the recovery of the final product being achieved by simply precipitating in water the thiourea **3** (waste economy), with only water and hydrochloric acid generated as by‐products of the reaction and with higher yield (93 %) compared to solution‐based procedures involving click‐type amine‐isothiocyanate coupling reaction (62–70 %).[^40^, ^41^]

### Preparation of BLG protein : hydantoin hybrids

**Scheme 2:**
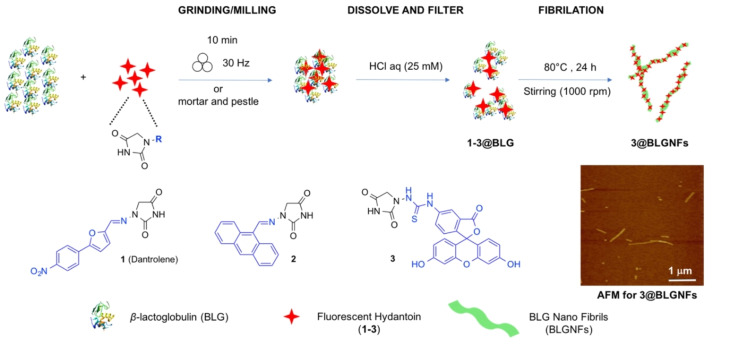
Schematic illustration of preparation of β‐lactoglobulin materials functionalized with hydantoin compounds: **1**–**3**@BLG and **3**@BLGNFs, including the AFM image for **3**@BLGNFs.

The fluorescent hydantoins **1**–**3** were incorporated into BLG and BLGNFs by a combination of mechanochemistry and aqueous self‐assembly, leading to protein : hydantoins **1**–**3**@BLG and **3**@BLGNFs, as outlined in Scheme 2. In a typical experiment, the fluorescent dye (compounds **1**–**3**) was ground with BLG (typically 1 mg of either of **1**–**3** and 50 mg BLG were used) in a ceramic mortar and pestle continuously for 10 min. The resulting powder was dissolved in 25 mm hydrochloric acid and the resulting solution/dispersion was filtered through a 0.45 μm polyethersulfone (PES) filter, resulting in clear solutions that were investigated by absorption and fluorescence spectroscopy. In a parallel experiment, the powdered mixture of BLG protein and the fluorescent labelled hydantoin (compounds **1**–**3**) were horizontally ball‐milled at 30 Hz during 10 min, using 1.5 mL stainless‐steel jars containing 20 balls (3 mm in diameter with total weight of 0.65 g). Manual grinding by mortar and pestle or automated milling by mixer‐mill led to samples displaying identical spectroscopic properties and concentration of hydrophobic compounds embedded into the BLG protein. The BLG:hydantoin hybrids can either be employed as prepared, or BLG can be heated at 80 °C for 24 h in order to convert BLG into BLG‐PNFs (hereafter labelled as BLGNFs), resulting in BLGNFs:hydantoin hybrids.

**Figure 1:**
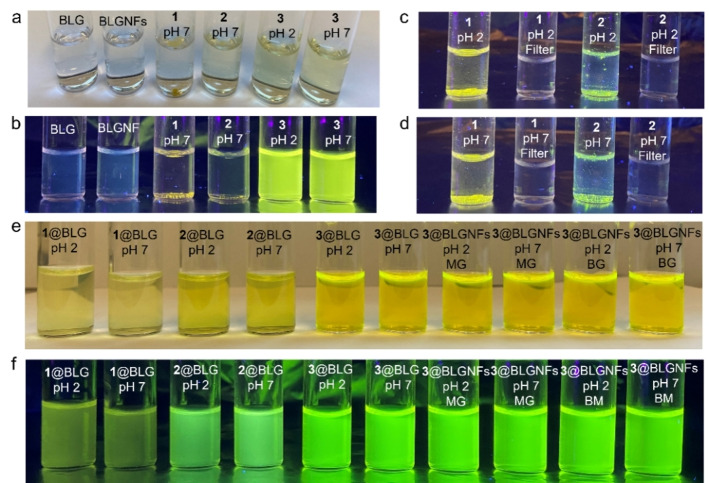
(a, b) Photographs of BLG, BLGNFs, **1**–**3**, **1**–**3**@BLG, and **3**@BLGNFs, in pH 2 and pH 7 buffer under ambient light and UV (365 nm) light. (c, d) Attempts at dispersing **1** and **2** in pH 2 and 7 buffer before and after filtration, under UV (365 nm) light. (e, f) Photographs of **1**–**3**@BLG and **3**@BLGNFs, prepared by manual grinding (MG) and Ball milling (BM), in pH 2 and pH 7 buffer, under ambient light and UV (365 nm) light.

It should be noted that hydantoins **1**–**3** have different solubilities in water (Figure 1). As shown in Figure 1a–d, hydantoins **1** and **2** were not soluble in either pH 2 or pH 7 buffer while hydantoin **3** is slightly soluble and displays a pH‐dependent solubility, with a higher solubility at higher pH values (0.05 mg mL^−1^ at pH 2 and 0.11 mg mL^−1^ at pH 7). This property is useful as pH thus may potentially be employed to trigger the release of compound **3**. The solubility properties for compound **3** at both pH 2 and 7 are illustrated in Figure 1a, b. Upon milling hydantoins **1**–**3** with BLG protein, the BLG helps the hydrophobic compounds to be dispersed in the buffer solutions. The maximum concentrations of hydantoins **1**–**3** dispersed with 10 mg mL^−1^ BLG in a pH 2 buffer solution were 0.12, 0.09, and 0.45 mg mL^−1^, respectively, while in a pH 7 buffer solution, the maximum concentrations were 0.12, 0.10, and 0.45 mg mL^−1^, respectively (Figure S6, Table 1). The maximum loading efficiencies of hydantoins **1**–**3** in BLG were thus 83, 67, and 90 % at both pH 2 and pH 7.

**Table 1:** Loading efficiency (LE) of hydantoins **1**–**3** (at BLG concentration 10 mg mL^−1^) and solubility (Sol.) of hydantoins **1**–**3**.

BR^[a]^ (pH)	1@BLG		2@BLG		3^[b]^	3@BLG	
	Sol. [mg mL^−1^]	LE [%]	Sol. [mg mL^−1^]	LE [%]	Sol. [mg mL^−1^]	Sol. [mg mL^−1^]	LE [%]
2	0.12	83	0.10	67	0.05	0.45	90
7	0.12	83	0.10	67	0.11	0.45	90

[a] Britton‐Robinson buffer. [b] 3 in Britton‐Robinson buffer (without BLG).

Mechanochemical treatment was essential to enable dispersion of **1** and **2** in water. In the absence of grinding, when **1** or **2** were added to a BLG aqueous solution, no hydantoin remained after filtration through a 0.45 μm PES filter. On the other hand, the presence of many polar groups makes **3** slightly soluble in water; however, complexation with BLG or BLGNFs enables a four times higher concentration to be achieved at pH 7 (0.45 vs. 0.11 mg mL^−1^) and a nine times higher concentration to be achieved at pH 2 (0.45 vs. 0.05 mg mL^−1^).

**Figure 2:**
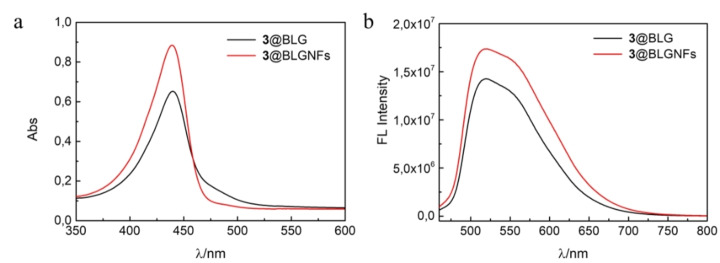
(a) UV/Vis spectra of β‐lactoglobulin functionalized with hydantoin **3**. (b) Fluorescence spectra of β‐lactoglobulin functionalized with hydantoin **3**.

In all cases when BLG:hydantoin hybrids were heated at acidic pH this resulted in formation of BLGNFs as demonstrated by atomic force microscopy (AFM) (see the Supporting Information, Figure S8). However, as explained in the Supporting Information, hydantoins **1** and **2** undergo hydrolysis during fibrillation (Figures S9 and S10). As **1** and **2** contain a slightly acid‐labile *N*‐acylhydrazone linker between the fluorescent moiety (generated from an aromatic aldehyde) and the hydantoin scaffold, the behavior of compound **3**, more stable towards hydrolysis, was investigated. Therefore, compound **3** was milled together with BLG protein as described above, and the resulting hybrid material was dissolved in water and then heated at acidic pH. This resulted in formation of BLGNFs (**3**@BLGNFs). In contrast to **1** and **2**, as shown in Figure 2, absorption and fluorescence spectra of protein mixed with hydantoin **3** does not significantly change before and after PNFs formation (see Figure 2 and Figures S9 and S10 in the Supporting Information). In addition, AFM images show typical fibrillar structures (shown in Scheme 1 and Figure S8d in the Supporting Information) consistent with previously reported fibrils from β‐lactoglobulins.^[43]^

### Structural characterization of BLG protein : hydantoin hybrids

In addition to spectroscopic characterizations of protein : hydantoin hybrids and AFM imaging of fibrillated samples, other investigations were carried out to determine the effect of hydantoins **1**–**3** on protein secondary structure (by circular dichroism spectroscopy), on colloidal properties (by zeta‐potential measurements), on size (by dynamic light scattering measurements) of proteins (both BLG and BLGNFs), and on protein : hydantoin hybrids (hybrids between BLG and compounds **1**–**3**, as well as hybrids between BLGNFs and compound **3**).

### Circular dichroism of BLG protein : hydantoin hybrids

**Figure 3:**
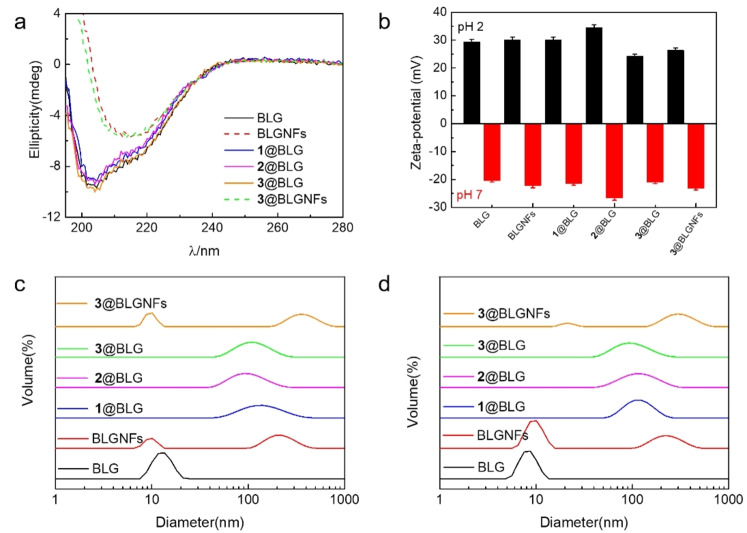
(a) Circular dichroism spectra of BLG, BLGNFs, and its complexes with hydantoins **1**–**3** at pH 2. (b) Zeta‐potential of BLG, BLGNFs, and its complexes with hydantoins (**1**@BLG, **2**@BLG, **3**@BLG, **3**@BLGNFs) at different pH values of 2 and 7. *T*=25 °C. (c, d) Volume size distribution of BLG and its complexes at 1 % *w*/*w* and different pH: (c) 2.0 and (d) 7.0. *T*=25 °C.

In order to investigate the effect of the presence of hydantoins on the secondary structure of the protein, BLG:hydantoin hybrids (with the protein either in the form of BLG or BLGNFs) the samples (along with non‐functionalized BLG and BLGNFs) were investigated by circular dichroism (CD) spectroscopy. In Figure 3a CD‐spectra of the various combinations are shown. In protein CD‐spectroscopy α‐helices typically display two absorptions located at 208 and 222 nm, whereas an absorption band at 217 nm is typical of random coil/β‐sheet secondary structure.^[44]^ When comparing the CD spectrum of BLG with the spectra of that BLG functionalized with **1**–**3**, the spectra are essentially identical, meaning that the presence of **1**–**3** does not significantly perturb BLG secondary structure. By comparison, when the native state BLG protein is converted into BLGNFs the CD spectra changes dramatically with a loss of absorptions typical of α‐helix, and the appearance at 217 nm typical of random coil/β‐sheet secondary structure. The CD spectrum of the **3**@BLGNFs sample also has a minimum around 217 nm, again indicating that the presence of **3** does not significantly perturb the secondary structure in BLGNFs.

### Zeta‐potential and size distribution measurements of BLG protein : hydantoin hybrids

From a delivery perspective, an interesting aspect of proteins materials is their ability to display negative or positive net charges, depending on the pH. At the pI the net charge of the protein will be zero, whereas below pI the net charge will be positive and, conversely, above pI the net charge will be negative. This provides the possibility of designing drug delivery systems, where as a result of pH modification a host and guest acquire the same net charge with more rapid release of the guest as a result of electrostatic repulsion. In the context of this study, such effects may be relevant for systems involving hydantoin **3**, as at higher pH the compound will be deprotonated and acquire negative charge, while at the same time at higher pH the BLG protein will also acquire a net negative charge. In addition, proteins and protein fibrils are colloidal systems, and an important parameter in determining the colloidal stability of such systems is their net charge, as they are electrostatically stabilized.

The zeta‐potentials of the different samples including BLG, BLGNFs, and their complexes with compound **1**–**3** were determined to study the effect of the presence of the hydantoins on the zeta‐potential of the protein materials (Figure 3b). Measurements were made at pH 2 and pH 7. At pH 2, which is lower than the isoelectric point (pI=5.1) of BLG,^[45]^ both BLG and BLGNFs have a positive zeta‐potential. Functionalized BLG and BLGNFs also have positive zeta‐potentials at pH 2, with a slightly more positive zeta‐potential for BLGNFs (30 mV) compared to BLG (29 mV). At pH 7 (higher than the BLG pI=5.1) both BLG and BLGNFs, as well as the corresponding functionalized materials have a negative zeta‐potential. This is important in the case of **3**, as at pH 7 (as pointed out earlier) this compound will be negatively charged, and the resulting electrostatic repulsion between protein and **3** may influence the release properties when the proteins are incorporated into drug delivery system.

Dynamic light scattering (DLS) can be employed to investigate the size distributions of colloidal particles, and the different materials were investigated at both pH 2 and pH 7. Figure 3c,d shows the various volume size distributions. For BLG there is a maximum value about 10 nm, which corresponded to the dimeric form of BLG. After formation of BLGNFs, there appeared a new peak at 200 nm, indicating the formation of larger particles. When comparing the size distribution of BLG and BLG functionalized with **1**–**3**, it is found that the size of the functionalized BLG particles is larger and is increased to about 100 nm. As all the **1**–**3** are hydrophobic and have a low solubility in water, there will be interactions between hydantoins and BLG, where BLG help the **1**–**3** dispersed in the aqueous environment. The protein accordingly acts as a surfactant that helps to disperse hydrophobic **1**–**3**. Comparing the BLGNFs and **3**@BLGNFs samples, in the case of **3**@BLGNFs the new peak appears at 400 nm (compared to 200 nm for BLGNFs), indicating the formation of larger particles in the presence of **3**. Figure 3d shows the corresponding DLS measurements performed at pH 7. The results of samples at pH 7 showed the same trends as for samples measured at pH 2.

### Protein : hydantoin hybrids hydrogels for drug delivery

One approach for delivery of hydrophobic drugs is the utilization of hydrogels.^[46]^ Although a large majority of the investigations of hydrogels for drug delivery focus on hydrophilic drugs, there has been much recent interest in the application of hydrogels for delivery of hydrophobic molecules. In order to enable delivery of hydrophobic substances a variety of approaches have been developed. These include: (a) direct incorporation of the hydrophobic molecule within the hydrogel, (b) adding drug loaded particles into the hydrogel, and (c) various methods where a solution of the drug in organic solvent is employed to add the drug to the hydrogel. Within the context of the present study, we have employed methods (a) and (b). In approach (a), **3** has been directly added to the gel (as a control in order to evaluate the effect of the presence of protein), and in approach (b) BLG‐particles (either BLG or BLGNFs) loaded with **1**–**3** were added to the gel. The protein particles loaded with **1**–**3** were prepared employing the combination of mechanochemistry and self‐assembly outlined previously. Note that, as mentioned earlier, **1** and **2** were unstable under the conditions employed for fibrillation, only **1**@BLG and **2**@BLG were incorporated into gels. In the case of **3**, both **3**@BLG and **3**@BLGNFs have been incorporated into gels.

### Preparation of gels

**Figure 4:**
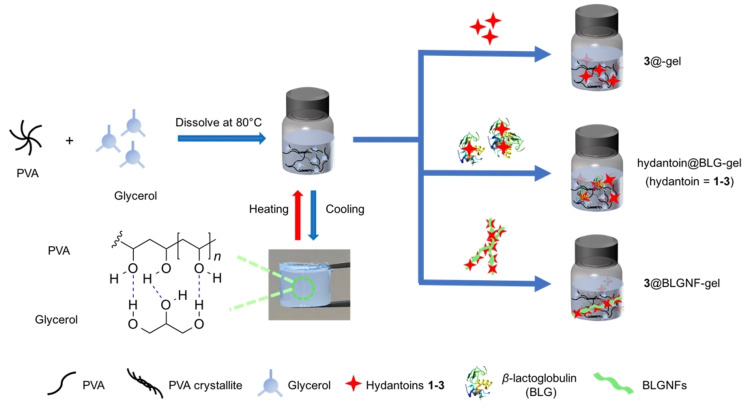
Schematic illustration of the preparation of PVA‐hydantoin, PVA‐BLG‐hydantoin, and PVA‐BLGNFs‐hydantoin hydrogels.

PVA was employed as base material for the preparation of hydrogels and incorporation of the protein : hydantoin materials. PVA is a widely used material due to its biocompatibility and biodegradability.[^47^, ^48^] An attractive option for preparing PVA‐hydrogels is to employ glycerol as a cross‐linking agent. Glycerol is a clear, colorless, odorless, viscous, and hygroscopic liquid obtained as byproduct in biodiesel production.^[49]^ Glycerol has been employed for a wide range of applications in the food industry (e. g., plasticizer, stabilizer, and emulsifier), as a humectant in cosmetic formulations,[^49^, ^50^] and as green solvent in organic synthesis.^[51]^ PVA/glycerol hydrogels have been investigated as an insulin release template for the treatment of diabetes.^[52]^ Figure 4 schematically shows the preparation of the PVA/glycerol‐protein : hydantoin hydrogels employed herein. Briefly, a solution of PVA in a glycerol: water mixture (in a 3 : 2 ratio, *v*/*v*) is heated at 90 °C for 30 min in order to turn the gel into a sol (see the Supporting Information). Then, the different materials (either **3** or BLG complexes **1**@BLG, **2**@BLG, **3**@BLG, or **3**@BLGNFs‐gel, or **3**‐gel for the reference gel, without added protein).

### Mechanical properties of protein : hydantoin hybrids hydrogels

**Figure 5:**
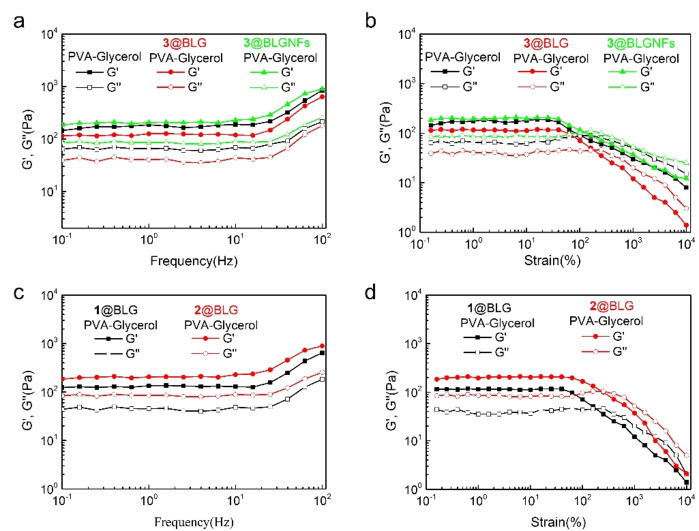
Rheological measurements of dynamic frequency sweep of (a) the PVA/glycerol and its complexes (BLG and BLGNFs functionalized with **3**) gels at a strain of 1 % over a range of 0.1–100 Hz, and (b) the PVA/glycerol and its complexes (BLG and BLGNFs functionalized with **3**) gels at a constant frequency of 1 Hz over a strain range of 0.1–10000 %. (c) BLG functionalized with **1** and **2** gels at a strain of 1 % over a range of 0.1–100 Hz. (d) Dynamic strain sweep of the BLG functionalized with **1** and **2** gels at a constant frequency of 1 Hz over a strain range of 0.1–10000 %.

For future applications in drug delivery, it is valuable to know the effect of incorporation of protein particles on the mechanical properties of the PVA/glycerol gels. Accordingly, a rheometer was employed to evaluate the mechanical viscoelastic properties of the gels with and without added BLG or BLGNFs (Figure 5). In a rheometer, the sample is allowed to gel between two plates. The bottom plate is stationary whereas the top plate can undergo angular motion, resulting in a (mechanical) shear stress and deformation of the gel. Data analysis of the resulting stress and strain curves will provide information regarding the storage modulus (*G*′, describing the solid, elastic character of the gel) and loss modulus (*G*′′, describing the liquid viscous character of the gel). In Figure 5a data for the variation of *G*′ and *G*′′ as a function of frequency (high frequency corresponding to rapid movement of the top plate, meaning that the gel is rapidly deformed) are provided for a reference PVA/glycerol gel. At all frequencies, *G*′>*G*′′, demonstrating the solid‐like character of the gels. Up to a frequency of about 30 Hz both *G*′ and *G*′′ are constant; however, at frequencies >30 Hz there is an increase in both moduli. When **3**@BLG was added to the gel, resulting in a **3**@BLG‐gel, a decrease in both *G*′ and *G*′′ was observed, in comparison with the reference gel. On the other hand, upon addition of **3**@BLGNFs, resulting in a **3**@BLGNFs‐gel, there was a slight increase in both *G*′ and *G*′′ compared to the reference gel. However, the trends regarding variation of *G*′ and *G*′′ as a function of frequency were the same for all the three gels. The effect of variations in strain was then investigated for the different gels (Figure 5b). For all the investigated gels, *G*′>*G*′′ at low strain. However, in the case of the PVA/glycerol gel (that is, a control sample without any added protein) at a strain of about 100 %, *G*′′>*G*′ meant that the viscous properties were dominant, and the gel broke. For both gels with added protein (**3**@BLG‐gel and **3**@LBGNFs‐gel) *G*′>*G*′′ for strains lower than 200 %, whereas the gel broke when *G*′′>*G*′ was above 200 %. The addition of protein accordingly seems to allow the gel to keep its solid‐like character at more severe deformations, compared to the PVA/glycerol reference gel (without added protein). In addition, compared to the reference gel, hydrogels containing BLGNFs exhibited increased moduli, indicating that the presence of fibrils makes the gels stiffer. On the other hand, hydrogels with BLG exhibited decreased moduli (Figure 5a) for gels incorporating **3**@BLG. One possible explanation is that the protein molecules disturb the cross‐linking between PVA and glycerol. In Figure 5c,d the data of variations of rheological properties for gels incorporating compound **1**@BLG and compound **2**@BLG are presented. The trends relative to the reference gel (in Figures 5a,b) were similar to those observed for **3**@BLG. In summary, the gels do not suffer of any dramatic negative effects on the mechanical properties due to the addition of proteins and can be considered for applications involving release of bioactive molecules.

### Release efficiency of hydantoins *in vitro*

**Figure 6:**
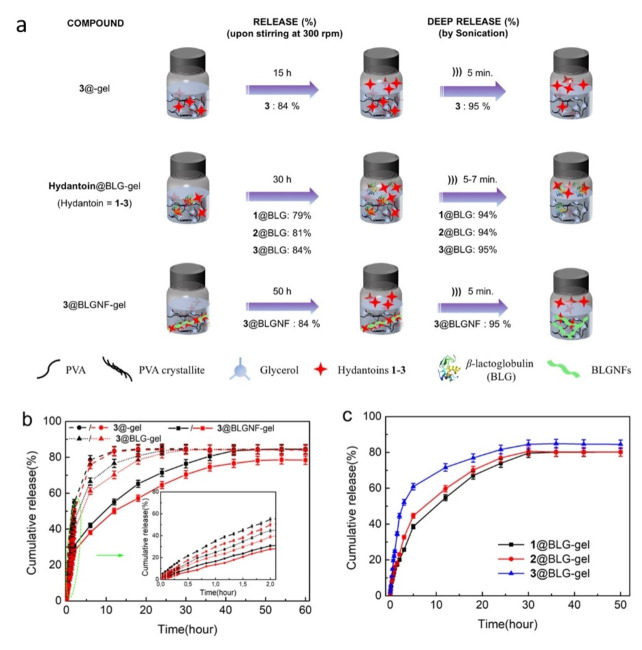
(a) Schematic drawing of release experiments from **1**@BLG‐gel, **2**@BLG‐gel, **3**@gel, **3**@BLG‐gel, and **3**@BLGNFs‐gel; the value in parentheses corresponds to experiments performed at pH 7. (b) Cumulative drug‐gel release of profiles at pH 2 (red curve) and 7 (black curve). Inset is the release within the initial 2 h. (c) Release profiles of **1**–**3**@BLG at pH 7.

In order to test the release of hydantoins **1**–**3** from the gels, experiments were performed with gels incorporating BLG or BLGNFs combined with **1**–**3** (Figure 6a). The experiments involving compound **3** are discussed first. Hydantoin **3** is slightly soluble in water, and its solubility is pH dependent. The release of **3** from hydrogels containing **3**@BLG (labelled as **3**@BLG‐gel) and **3**@BLGNFs (labelled as **3**@BLGNFs‐gel) was studied at two different pH (2 and 7) over a time course of 60 h (at room temperature with stirring at 300 rpm) by inserting the gel into Britton‐Robinson (BR) buffer and then removing samples at regular time (1 h) intervals for analysis by spectroscopy. To determine the effect of the presence of the protein materials, control experiments were also performed by adding compound **3** to the gel (labelled as **3**‐gel, that is a gel without any added protein). During the initial 2 h (see the inset of Figure 6b) the release rate displayed a linear profile in all cases. After 2 h, the amount of **3** released from **3**‐gel corresponded to 55 % in pH 7 buffer, and 50 % in pH 2 buffer. The **3**@BLG‐gel displayed a 44 % release at pH 7 and 39 % at pH 2. The **3**@BLGNFs‐gel showed the slowest release, with 30 % released at pH 7 buffer and 28 % at pH 2. After this initial phase, the release rate slows down for all three systems (**3**@gel, **3**@BLG‐gel, **3**@BLGNFs‐gel), with the most pronounced retardation for **3**@BLGNFs‐gel, which may be due to the stronger association between **3** and BLGNFs. The gradual retardation of the release rate is most likely due to the establishment of an equilibrium between adsorption of **3** onto the BLGNFs surface, and desorption and release of surface‐bound **3** into the surrounding media. The **3**‐gel reaches equilibrium after 15 h and **3**@BLG‐gel reaches equilibrium after 30 h with an 84 % release in both cases (at both pH 2 and pH 7). In the case of BLGNFs the system had not yet reached equilibrium even after 30 h. After 30 h the **3**@BLGNFs‐gel displayed a 76 % release at pH 7, and a 71 % release at pH 2. Equilibrium was reached after 50 h, with the **3**@BLGNFs‐gel displaying a release of 84 % at pH 7 and 78 % at pH 2. For all systems involving **3**@BLG or **3**@BLGNFs the release rate is slightly higher at pH 7 compared to pH 2, with a likely explanation being electrostatic repulsion between the negatively charged protein and negative charges on compound **3** due to deprotonation of phenolic protons. These results indicate that the possibility of converting BLG into BLGNFs may be useful in tuning the properties of BLG‐based delivery systems.

After being kept for in total 60 h at room temperature with 300 rpm stirring at either pH 2 and 7, we tested if remaining **3** could be induced to rapidly discharge from the gel by refreshing the buffer. When the gels were immersed in fresh BR buffer (pH 2 and 7) for 24 h, **3** was not released, maybe due to the compounds being entrapped into the gel (Figure S7a). However, when the vials were placed in an ultra‐sonication bath, and were sonicated for 10 min at ambient temperature, **3** was rapidly released within 5 min, reaching up to a total release of 95 % (at both pH 2 and 7) for **3**‐gel, **3**@BLG‐gel, and **3**@BLGNFs‐gel (Figure S7a, b). Finally, the gels were dissolved in water at 90 °C, and the resulting solutions were investigated by fluorescence spectroscopy confirming that around 5 % of **3** was left in the remaining samples for **3**‐gel, **3**@BLG‐gel, and **3**@BLGNFs‐gel.

In addition, the release properties of hydrophobic hydantoins (**1** and **2**) in BLG were also investigated at pH 7. The gels were inserted into BR buffer and were stirred at 300 rpm and samples were removed at regular time (1 h) intervals for analysis by spectroscopy. As shown in Figure 6c, the release rate of hydrophobic hydantoins from **1**@BLG‐gel and **2**@BLG‐gel was lower than the release rate of the more hydrophilic **3**, as expected, considering that **3** is slightly water soluble, whereas, in the case of **1** and **2**, the diffusion from the gel required the BLG mediation. After about 30 h, equilibrium was reached: the **1**@BLG‐gel showed a 79 % release, while a release of 81 and 84 % was displayed respectively for the **2**@BLG‐gel and the **3**@BLG‐gel, respectively.

After being kept at room temperature with stirring 300 rpm for in total 36 h, we tested if also **1** and **2** could be induced to rapidly discharge from the gel by refreshing the buffer or applying sonication. When the gels were immersed in fresh BR buffer (pH 7) for 24 h, **1** and **2** were not released, probably because the compounds were entrapped in the gel (Figure S7b). However, when the gel was in pH 7 buffer and the vial was sonicated for 10 min, a 94 % (rapid) release occurred already after 7 min for both the **1**@BLG‐gel, and the **2**@BLG‐gel (Figure S7b). Finally, the gels were dissolved in water at 90 °C and it was determined, by fluorescence spectroscopy, that around 6 % of **1**@BLG and **2**@BLG were left in the gels. The release data for **3**@BLG and **3**@BLGNFs suggested that manipulating the states of proteins can tune the drug release rate from gels. **3** added to BLG and in particular BLGNFs showed long and durable release profiles, compared to **3** without protein.

## Conclusions

Mechanochemical processes have the advantage that they do not require a solvent. This means that mixtures of hydrophobic and hydrophilic substances can be mixed efficiently in the solid state. In this regard, the water‐soluble protein β‐lactogobulin (BLG) was used to disperse fluorescently labelled hydrophobic hydantoins **1**–**3**. Milling of an excess of protein and the hydrophobic material leads to formation of a hybrid material, where the hydrophobic molecule/material is dispersed into the protein matrix. Upon addition of water, the protein acted as a surfactant, helping to disperse the hydrophobic materials **1**–**3** in water and resulting in **1**–**3**@BLG. In the case of **3**@BLG heat treatment lead conversion of BLG into nanofibrils, giving **3**@BLGNFs. The different protein : hydantoin materials can be incorporated into polyvinyl alcohol (PVA)/glycerol gels. When the release profiles of these different systems were compared, it was shown that the release rate of **3** followed the trend **3**>**3**@BLG>**3**@BLGNFs. The release raSte of **3** can in other words be: (a) tuned by structural modifications of the protein and (b) influenced by the pH of the surrounding medium. Indeed, at pH 7 the protein particles will have a negative net charge and electrostatic repulsion between **3** and the protein will then promote faster release. This is a very beneficial property of the drug delivery system as it may allow selective delivery at a local environment with high pH value. In addition to protein particles loaded with **3**, it was also demonstrated that compounds **1** and **2** can be incorporated into protein particles (**1**@BLG and **2**@BLG) that can be added into the hydrogels. When the release rates of **1**@BLG, **2**@BLG, and **3**@BLG were compared, it was shown that the release rate (at pH 7) followed the trend **3**@BLG >**1**@BLG ≈**2**@BLG. The faster release rate of **3**@BLG was due to a combination of the higher solubility of **3** alone (compared to **1** and **2**) and electrostatic repulsion between the negatively charged protein and negatively charged **3** (whereas **1** and **2** do not have any charge).

To the best of our knowledge, mechanochemical methodology has not been employed so far to prepare hybrid materials for drug delivery purposes. Presently, the mechanochemical preparation of protein : hydantoin hybrids for delivery of hydrophobic fluorescently labelled hydantoins was investigated, but the method could also find applications as probes to detect nitric oxide (NO) by imaging in living cells.^[53]^ Fluoresceine‐tagged aminohydantoin derivatives can be also used for the detection of antibiotic metabolites in muscular tissues^[54]^ or in food,^[55]^ broadening the potential applications of mechanochemistry in medical sector. In addition, the use of the developed protein particles is not limited to delivery of drugs but can, for example, be employed as components of smart food (delivery of nutrients) or release systems of pesticides.

## Experimental Section

### Chemicals and reagents

β‐Lactoglobulin (BLG), glycerol, boric acid, and concentrated HCl (1 m) were purchased from Sigma‐Aldrich. Polyvinyl alcohol powder (PVA, *M*_w_ 89000–98000, 99+% hydrolyzed), was purchased from Sigma‐Aldrich. Hydantoins **1**, **2**, and **3** were synthesized as described below in detail, while compounds **4** and **5** are commercially available. The Britton‐Robinson (BR) buffer solutions at pH 2 (composed of 3.647 g L^−1^ 85 % H_3_PO_4_, 2.234 g L^−1^ acetic acid, and 2.301 g L^−1^ H_3_BO_3_ with 0.558 g L^−1^ NaOH) and pH 7 (composed of 2.570 g L^−1^ 85 % H_3_PO_4_, 1.575 g L^−1^ acetic acid, and 1.622 g L^−1^ H_3_BO_3_ with 2.754 g L^−1^ NaOH) values were prepared. The buffer solutions were used to control the acidity by using a biotrode pH meter (Hamilton Bonaduz AG, Switzerland) to measure the pH. All reagents were used directly and did not require further purification, and doubly distilled water (18.2 Ω) was used throughout.

### Preparation of hydantoin derivatives 1–3

All reagents were commercially available. NMR spectra were recorded at room temperature with the appropriate deuterated solvent (DMSO‐d_6_). Chemical shifts (*δ*) of ^13^C NMR spectra are reported in ppm relative to residual solvent signals (DMSO in DMSO‐d_6_: *δ*=39.62 ppm for ^13^C). ^13^C NMR spectra were registered at 400 MHz, the samples were prepared by dissolving 10 mg of hydantoin in 0.7 mL of deuterated solvent. The identity of analytically pure final products **1** and **2** was assessed by comparison of their spectral data previously described in the literature.^[3g]^ The identity of compound **3** (not previously reported) was investigated by ^1^H NMR, ^13^C NMR, and Fourier‐transform FT‐IR spectroscopy, as well as by mass spectrometry [liquid chromatography (LC)/MS and high‐resolution (HR)MS] (see the Supporting Information for data and spectra; Figures S1–S5). For compound **3**, peaks for the fluorescein residue were attributed by comparison with the experimental spectral data reported in databases.[^56^, ^57^] FT‐IR spectra were obtained by a Perkin Elmer Spectrum 100 FT‐IR Spectrometer (ATR‐IR device) in reflectance mode. Measurements were performed directly on the powder. The powders were placed on the ATR‐IR plate and a constant pressure (i. e., force gauge was kept at 19 % of the scale) was applied to the powder to bring it into good contact with the ATR crystal. Band positions are reported in cm^−1^. HRMS analyses were performed by means of a Q‐TOF mass analyzer (Waters, 2001) with electron spray ionization (ESI) mode [tolerance=1.0 mDa; Double bond equivalents (DBE): min=−3.0, max=100.0; element prediction: off; number of isotope peaks used for i‐FIT: 3; monoisotopic mass, even electron ions 1979 formula(e) evaluated with 4 results within limits (up to 50 best isotopic matches for each mass). Melting points were measured in an open capillary on a Stuart SMP50 automatic melting point apparatus. The ball‐milling experiments involving preparation of **1** and **2** were performed in a Pulverisette P7 Classic line (Fritsch GmbH, Idar‐Oberstein, Germany) using 12 mL zirconium oxide jar (with 25 zirconium oxide balls, 5 mm Ø; *m*_ball_=0.410 g each, *m*_tot_=10.25 g) for preparing compounds **1** and **2**.^[3g]^ The identity of analytically pure final products **1** and **2** was assessed by comparison of their ^1^H NMR data previously described in the literature.^[3g]^ The preparation of compound **3** was performed in a P23 vibrational ball mill (Fritsch GmbH, Germany) using 10 mL zirconium oxide jar (3 zirconium oxide balls, 10 mm Ø, *m*_ball_=2.95 g each, *m*_tot_=8.85 g).

### General procedure for the preparation of compound 3

1‐aminohydantoin hydrochloride (46.7 mg, 0.308 mmol, 1.2 equiv.) and fluoresceine isothiocyanate isomer I (100.0 mg, 0.257 mmol, 1.0 equiv.) were ball‐milled during 2 h 30 min at 50 Hz. The final product was recovered by precipitation in water (50 mL) and vacuum filtration. The crude was dried in vacuum over P_2_O_5_ overnight. A deep orange solid (120 mg, 93 % yield) was obtained.

### Preparation of BLG protein : hydantoin hybrids

In a typical procedure, a mixture of 1 mg of either hydantoins **1**–**3** or compounds **4** and **5** and 50 mg of BLG was ground continuously for 10 min with manual grinding by a ceramic mortar and pestle. After grinding, the resulting powders were dissolved in 5 mL of 25 mm hydrochloric acid followed by filtration through a 0.45 μm PES filter, separately, giving **1**–**5**@BLG dispersions. When the dispersions **1**–**5**@BLG was heated at 80 °C for 24 h with magnetic stirring (1000 rpm), PNFs were formed resulting in **3**@BLGNFs, **4**@BLGNFs, and **5**@BLGNFs, whereas the attempt at forming **1**@BLGNFs and **2**@BLGNFs resulted in hydrolysis of **1** and **2**. For preparations involving ball milling, hydantoin **3** (1 mg) and BLG (50 mg) were ball‐milled at 30 Hz for 10 min, with a mixer mill (MM 400, Retsch, Germany) using 1.5 mL stainless‐steel jars (with 20 stainless‐steel balls, 3 mm Ø; *m*_ball_=0.033 g each, *m*_tot_=0.65 g). After milling, the resulting powder was treated as described above for the sample prepared by manual grinding.

### Characterization of BLG protein : hydantoin hybrids

#### Absorption spectroscopy

The hybrids between hydantoins and BLG or BLGNFs were studied by measuring the absorbance changes of hydantoins and hydantoin protein complexes by employing a UV‐2450 spectrophotometer (Shimadzu, Japan). To verify the decomposition of hydantoin **1** and **2** during PNFs formation, hydantoins **1**, **2**, **4**, and **5** and their respective protein matrix samples were diluted 10 times with 25 mm HCl prior to measurement of absorbance spectra (Figure S9). The solubility of hydantoins **1**–**3** and their protein matrix were evaluated by measuring the absorbance at 390, 460, and 440 nm, respectively (Figure S6).

#### Fluorescence spectroscopy

To verify the decomposition of hydantoin **1** and **2** during PNFs formation, the hydantoins and their protein matrix samples were diluted 100 times with 25 mM HCl, and then the fluorescence was checked with a fluorescence spectrofluorometer (Horiba Jobin‐Yvon Fluoromax‐4) and the entrance and exit slits were set at 5 nm. The emission spectra of hydantoin **1** and **4** were measured with an excitation wavelength of 350 nm, while hydantoin **2** and **5** were excited at 370 nm (Figure S10). For the investigation of hydantoin release *in vitro*, the fluorescence intensity at 501, 526, and 520 nm of **1**–**3**@BLG and **3**@BLGNFs were recorded and the release of hydantoins in the supernatant was determined by the relative fluorescence intensity (corrected for dilution effects and compared to the intensity of the hydantoin: protein complex added to the gel) (Figure 6 and Figure S7). The samples with hydantoins **1**–**3**@BLG and **3**@BLGNFs were excited at 390, 460, and 440 nm, respectively.

#### Atomic force microscopy

The morphology of native BLG, BLGNFs, and their complexes with hydantoins was studied using AFM (digital instruments dimension 3100 atomic force microscope). Different specimens were diluted to a final concentration of 0.001 mg mL^−1^; the diluted sample (20 μL) was drop casted on a silicon slide and left for 1 min, before a nitrogen gun was used to remove excess sample.

#### Circular dichroism spectra

CD spectra were recorded by using a Jasco J‐715 spectropolarimeter (Jasco, Tokyo, Japan). Far‐UV (190–280 nm) spectra of different samples with a final protein concentration of 0.1 mg mL^−1^ were recorded in 0.01 cm path length cell (Hellma, Müllheim/Baden, Germany) using a step size of 0.5 nm, bandwidth of 1 nm, and scan rate of 50 nm min^−1^. Each protein spectrum was obtained by averaging 10 scans and corrected by subtracting the solvent spectrum at room temperature.

#### Zeta‐potential and size distribution measurements

The zeta‐potential and size distribution of different sample solutions including BLG, BLGNFs, and their complexes with hydantoins at pH value of 2 and 7 were determined by Zetasizer Nano ZS (Malvern Instruments, UK). The zeta‐potential measurement was performed in a disposable capillary cell (DTS1070 Cell) employing a glass cuvette (10 nm slit). Every measurement was repeated three times with 10–100 runs at 25 °C after temperature equilibration for 120 s.

#### Loading efficiency of BLG protein : hydantoin hybrids

The loading efficiency (LE) of hydantoins **1**–**3** in BLG matrix were calculated by the following Equation 1:

(1) $$LE\left(\%\right)=W_{H}/W_{TH}$$

where *W*_H_ is the maximum weight of loaded hydantoins **1**–**3** in the supernatant and *W*_TH_ is the total weight of hydantoins **1**–**3**.

### Hydantoins release *in vitro*

Gels were prepared as follows: 1.8 g PVA was first dissolved in 20 mL of a mixture of glycerol (gly) and water (12 mL glycerol and 8 mL H_2_O) at 90 °C for 30 min. Then 0.9 mL of the PVA solution was mixed with 0.1 mL of BLG protein : hydantoin hybrids (**1**@BLG, **2**@BLG, **3**@BLG, **3**@BLGNFs) under stirring until a homogeneous solution was formed. The solution was then cast into mould (inner diameter 5 mm) and cooled at room temperature. In this manner, a series of hydrogels was prepared labelled as **1**@BLG‐gel, **2**@BLG‐gel, **3**@BLG‐gel, and **3**@BLGNFs‐gel. The hydrogels were added into 4 mL of pH 2 or pH 7 buffer solution with 300 rpm stirring and kept under ambient conditions. In order to determine the effect of protein, a reference gel, containing **3** without added protein, was prepared by mixing 0.1 mL of **3** dissolved in buffer (either at pH 2 or pH 7) with 0.9 mL of the PVA solution. The sample was then treated in the same manner as the others with protein.

The release from the gels was investigated by inserting the gel into BR buffer and then removing samples at regular time intervals for analysis. After the release had reached equilibrium, fresh buffer was added which resulted in no additional release; however, when the gel in buffer was sonicated during 10 min. (Elma Ultrasonics‐S15H, Germany) this resulted in additional release.

The analysis of removed samples was performed as follows: at the time point of analysis, 10 μL of sample was removed from the release system (and the system was refilled with an equal amount of the appropriate buffer) and diluted by 990 μL of pH 7 buffer. This was followed by recording of the fluorescence intensity. The release [%] was calculated by the Equation 2:

(2) $$release=F_{t}/F_{i}\;$$

where *F*_t_ is the fluorescence intensity of hydantoins released at the specific time point, and *F*_i_ is the initial fluorescence intensity of hydantoins added in the hydrogels. All samples were measured in triplicate at each time point.

## Conflict of interest

The authors declare no conflict of interest.

## Supporting information

As a service to our authors and readers, this journal provides supporting information supplied by the authors. Such materials are peer reviewed and may be re‐organized for online delivery, but are not copy‐edited or typeset. Technical support issues arising from supporting information (other than missing files) should be addressed to the authors.

Supporting Information
