# Supplementary material for: Mechanochemical Preparation of Protein : hydantoin Hybrids and Their Release Properties
Source: ChemSusChem. 2021 Dec 9;15(2):e202102097. doi: 10.1002/cssc.202102097 (PMC9299789; doi:10.1002/cssc.202102097)
Supplement: Supplementary file 1 — Supporting Information [file CSSC-15-0-s001.pdf]

ChemSusChem

Supporting Information

Mechanochemical Preparation of Protein : hydantoin Hybrids and Their Release Properties

Yusheng Yuan, Lei Wang, Andrea Porcheddu, Evelina Colacino,* and Niclas Solin*This publication is part of a joint Special Collection of *Chemistry-Methods* and *ChemSusChem* including invited contributions focusing on "Methods and Applications in Mechanochemistry". Please visit to view all contributions.© 2021 The Authors. ChemSusChem published by Wiley-VCH GmbH. This is an open access article under the terms of the Creative Commons Attribution License, which permits use, distribution and reproduction in any medium, provided the original work is properly cited.

Experimental Part.

Experimental data for 3.

MP. 299 °C (blackening); ^{1}H **NMR** (400 MHz, DMSO-d_6) δ (ppm): 11.36 (s_{broad}, 1H, CONHCO), 10.35 (s_{broad}, 1H, S=C-NH-N), 10.16 (s_{broad}, 2H, OH), 9.88 (s_{broad}, 1H, S=C-NH), 8.25 (s, 1H, ArH), 8.03 (*pseudo* s, 1H, ArH), 7.23 (pseudo s, 1H, ArH), 6.74-6.45 (m, 6H, ArH), 4.34-3.90 (m, 2H, CH_2); **^{13}C APT NMR** (100 MHz, DMSO-d_6) δ (ppm): 169.9, 168.3, 159.5, 151.8, 140.4, 128.9, 112.6, 109.5, 102.3, 83.1; **IR** (cm^{-1}) ν: 3040 (NH), 1723 (C=O), 1585 (C=S), 1532, 1489 (N-C-N thiourea), 1457, 1364, 1304, 1237, 1208, 1172, 1117, 850, 664; **ESI-(+)** *m/z*: 505.1 $[M+H]^+$, 348.1; **ESI-(-)** *m/z*: 1007.1 $[2M-H]^-$, 503.1 $[M-H]^-$, 460.1, 371.1; **HRMS ESI-(+)** calcd for $C_{24}H_{17}N_4O_7S$ $[M+H]^+$ 505.818, found 505.815. **HRMS ESI-(-)** $C_{24}H_{15}N_4O_7S$ $[M-H]^-$ 503.0661, found 503.0667.

FT-IR Reference Spectra of Starting Materials and Final Product 3.

Acquisition conditions: background, then NS = 16.

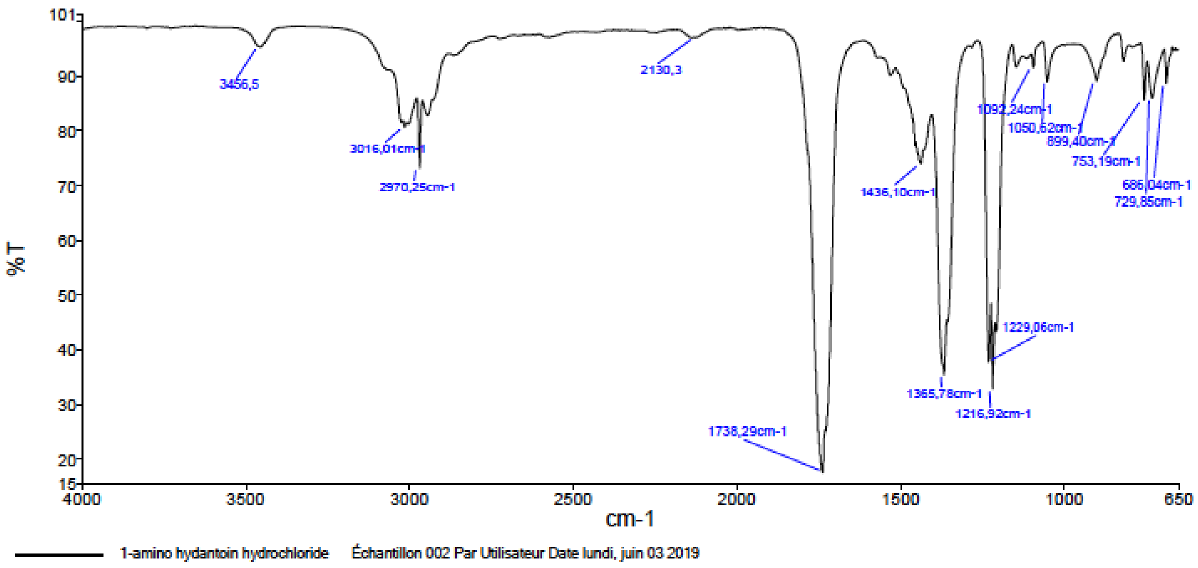

Figure S1. FT-IR spectrum of 1-aminohydantoin hydrochloride.

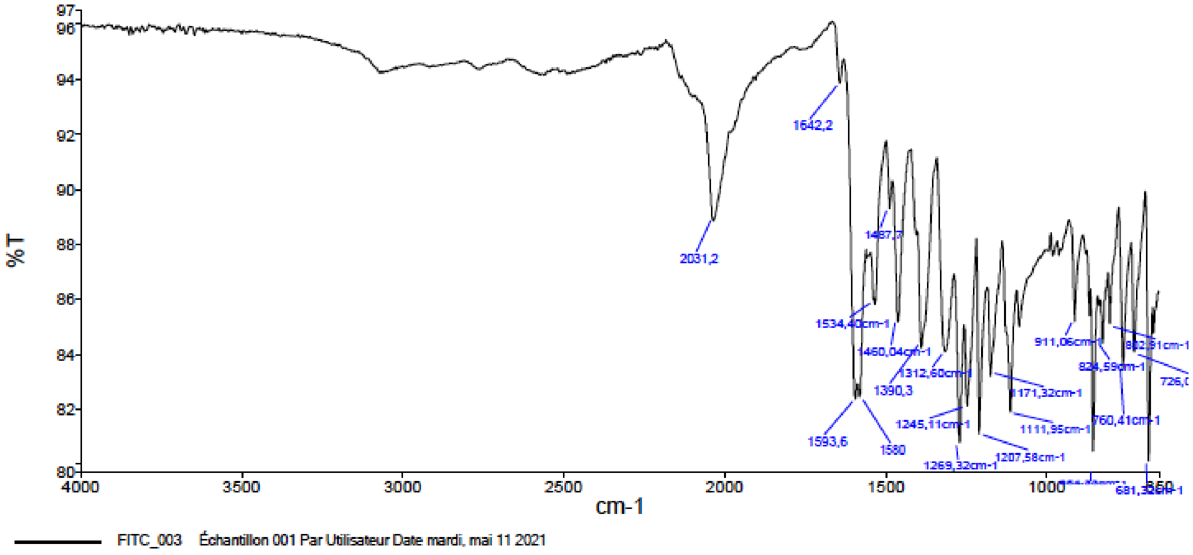

Figure S2. FT-IR spectrum of Fluoresceine Isothiocyanate (FITC).

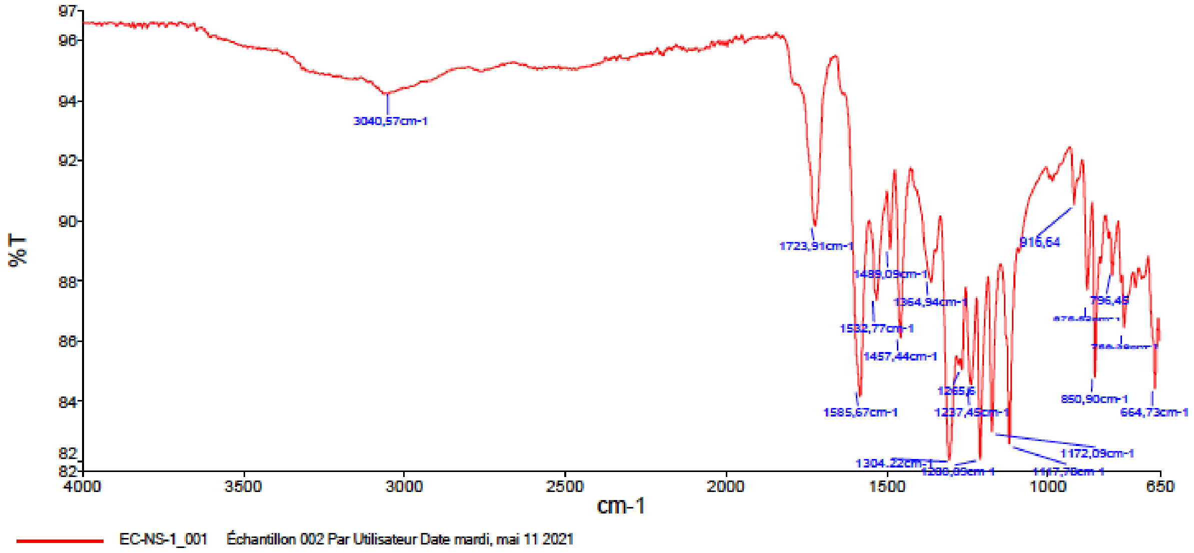

Figure S3. FT-IR spectrum of FITC-aminohydantoin **3** after work-up and prepared milling 1-aminohydantoin hydrochloride (1.2 equiv) and fluoresceine isothiocyanate (1.0 equiv.) during 150 min at 50 Hz, continuously.

NMR spectra of 3.

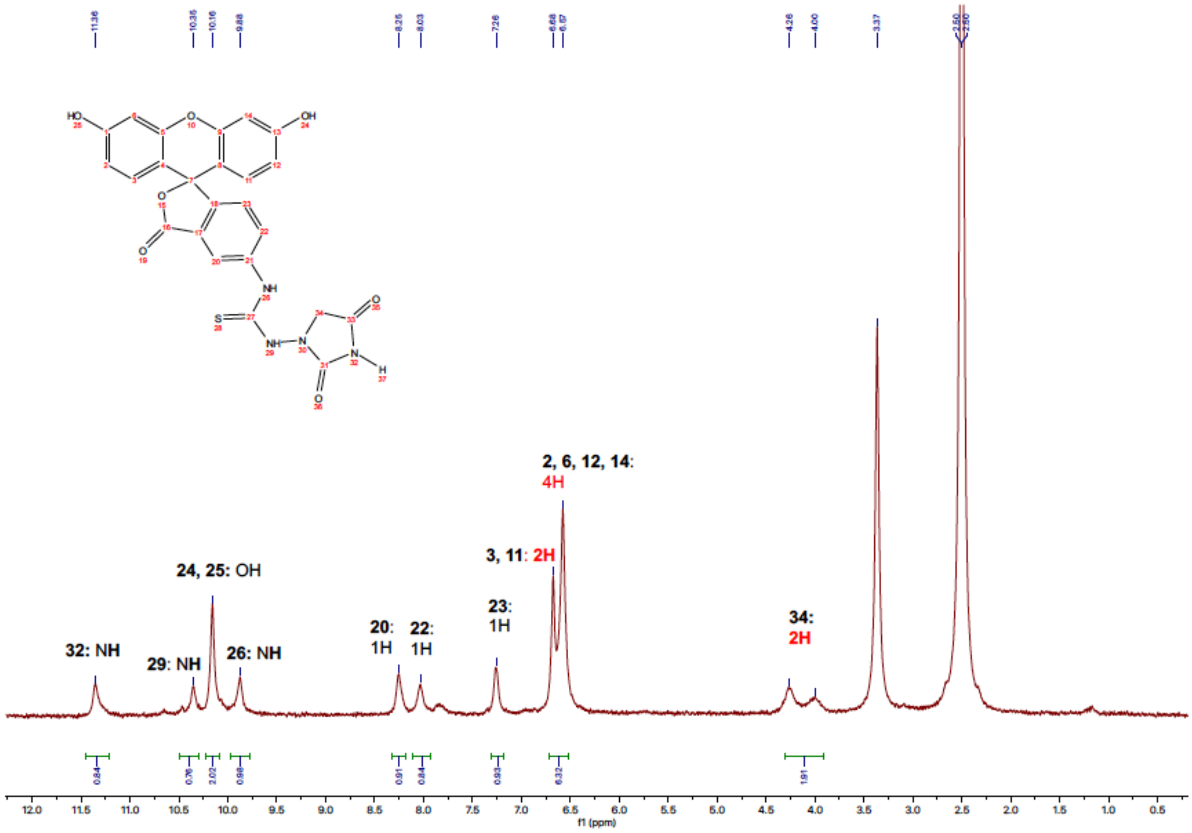

Figure S4. ^{1}H NMR spectrum of FITC-aminohydantoin **3.**

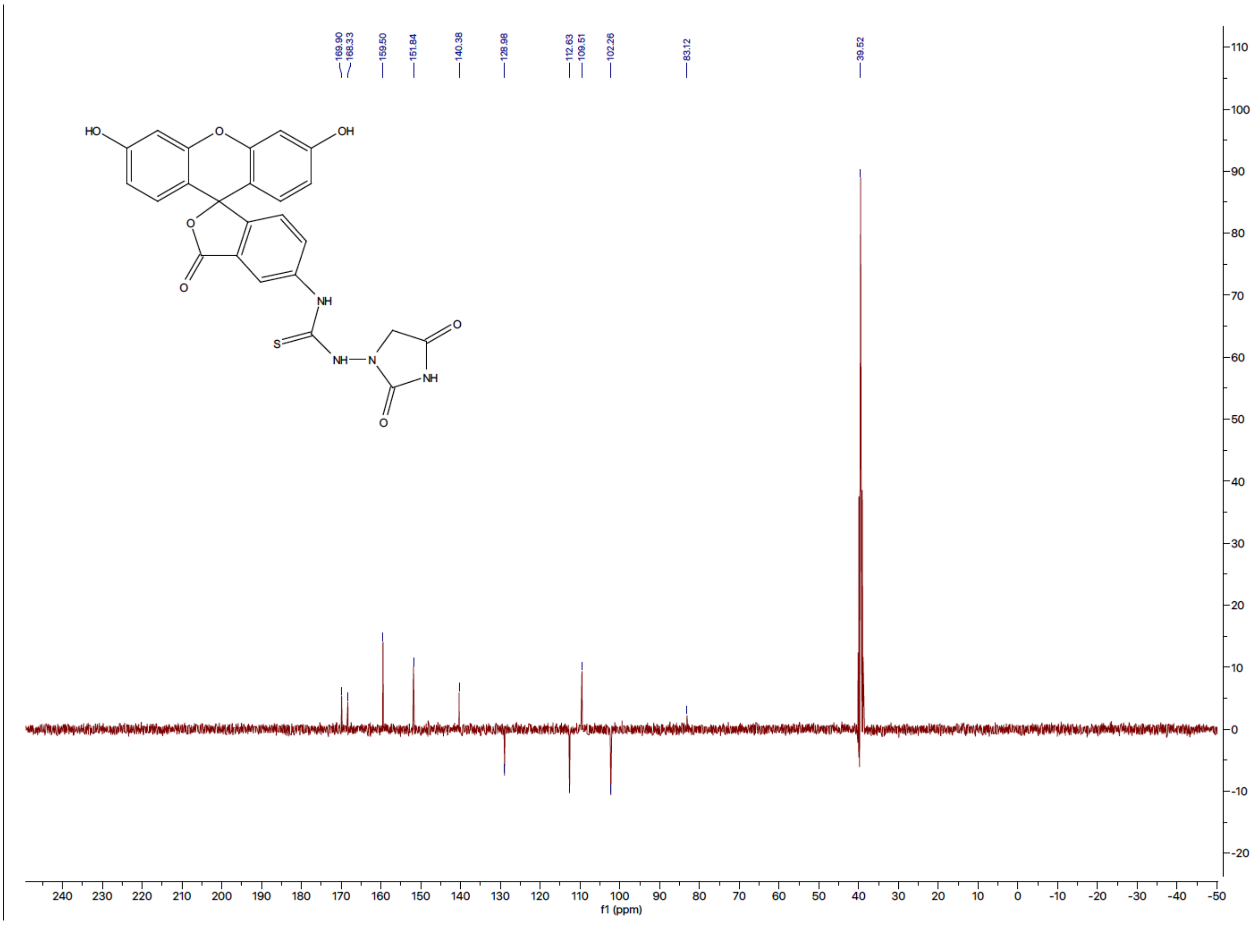

Figure S5. ^{13}C NMR spectrum of FITC-aminohydantoin **3.**

Determination of the Solubility of Hydantoins (1-3) and their protein complexes.

In order to determine the solubility of **3** at pH 2, different amounts of **3** (between 0.02-0.3 mg in 0.05 mg intervals) was weighted and mixed with 2 mL of pH 2 buffer. The resulting sample was left standing for 12 hours in order to allow undissolved particles to settle, and then the absorbance at 440 nm was measured. In order to determine the solubility of **3** at pH 7, 3 (0.02-0.3 mg in 0.05 mg intervals) was dissolved in pH 7 buffer. The resulting sample was left standing for 12 hours in order to allow undissolved particles to settle, and then the absorbance at 440 nm was measured. In addition, hydantoin **1** (0.1 mg) and **2** (0.1 mg) were weighted and added to pH 2 and 7 buffer separately, and hydantoin **1** (0.1 mg) and **2** (0.1 mg) were weighted and dissolve in pH 2 and 7 buffer followed by filter with 0.45 μm PES filter for comparison.

For samples involving proteins, first, hydantoin **1** (0.05-0.3 mg in 0.05 mg intervals), hydantoin **2** (0.05-0.3 mg in 0.05 mg intervals), hydantoin **3** (0.05-1 mg in 0.1 mg intervals) were mixed with 20 mg of BLG protein, and the mixture was ground continuously for 10 minutes in a ceramic mortar. The resulting powder was dissolved in 2 mL of BR buffer then the solutions were left standing for 12 hours in order to allow undissolved particles to settle prior check the absorbance at 390 nm, 460 nm, 440 nm separately (Figure S6). While for the pH 7 buffer, hydantoin **1** (0.05-0.3 mg with 0.05 interval), hydantoin **2** (0.05-0.3 mg with 0.05 interval), hydantoin **3** (0.05-1mg with 0.1 interval) were weighted and ground in a mortar with 20 mg of BLG protein by continuing grinding for 10 minutes. The powders were dissolved in 2 mL of pH 7 buffer separately, then the solutions were left standing for 12 hours in order to allow undissolved particles to settle prior check the absorbance. For the ball milling conditions, hydantoin **3** (0.05-1 mg with 0.1 interval) and BLG was ball-milled at 30 Hz for 10 minutes, employing 1.5 mL stainless steel jars, each equipped with 20 stainless steel balls (3 mm in diameter with total weight of 0.65 g). After milling the resulting powder was dissolved in 2 mL of pH 2 and 7 buffer, then the samples were left standing for 12 hours in order to allow undissolved particles to settle prior check the absorbance at 440 nm.

Solubility of Hydantoins (1-3) in water

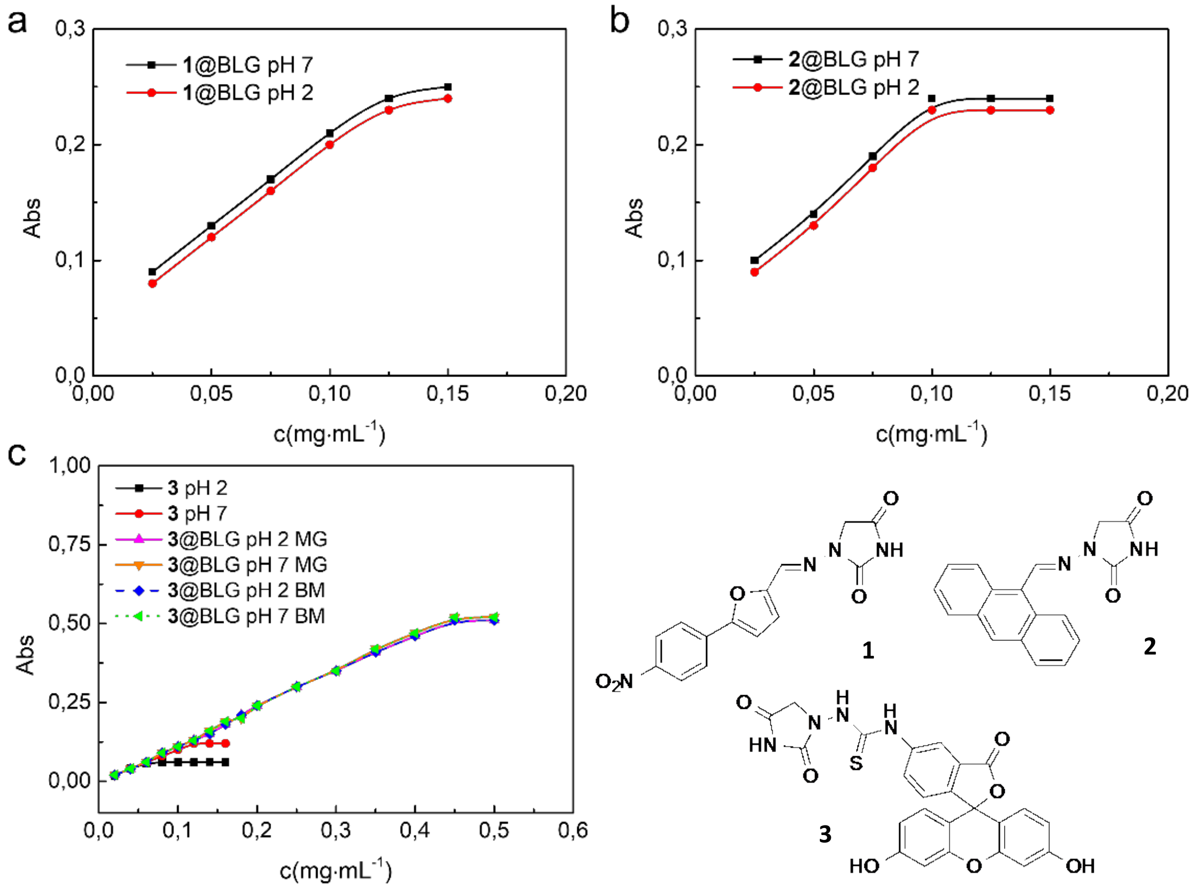

Figure S6. (a) Absorbance of hydantoin **1**@BLG matrix at pH 2 and 7 buffer among the range of 0.025-0.15 mg·mL^{-1}. (b) Absorbance of hydantoin **2**@BLG matrix at pH 2 and 7 buffer among the range of 0.025-0.15 mg·mL^{-1}. (c) Absorbance of hydantoin **3** and its BLG matrix upon manual grinding (MG) or ball milling (BM) at pH 2 and 7 buffer among the range of 0.025-0.5 mg·mL^{-1}.

Hydantoin Release in Vitro induced by sonication.

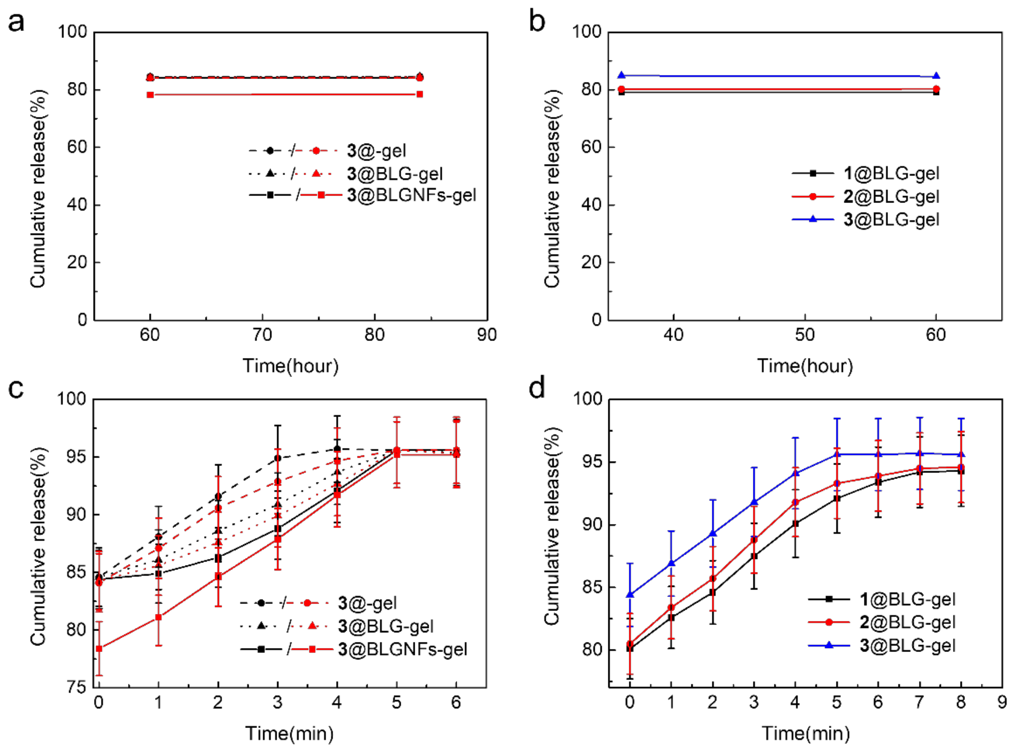

Figure S7. (a) Release profiles of samples **3**-gel, **3**@BLG-gel, and **3**@BLGNFs-gel at pH 2 (red line) and 7 (black line) for 24 hours. Before replacing the fresh BR buffers, samples were kept at pH 2 and 7 during 60 hours (as in **Figure 7b**) resulting in release of 76-85% of **3**. (b) Release profiles of samples **1-3**@BLG-gel at pH 7 in fresh BR buffers for 24 hours. Before replacing the fresh BR buffers, the samples were kept at pH 7 during 36 hours (as in **Figure 7d**) resulting in release of 79-84% of **1-3** prior to sonication. (c) Release profiles of samples **3**-gel, **3**@BLG-gel, and **3**@BLGNFs-gel at pH 2 (red line) and 7 (black line) under sonication for 6 minutes. Before sonication the samples were kept at pH 7 during 60 hours (as in **Figure 7b**) resulting in release of 76-85% of **3** prior to sonication. (d) Release profiles of samples **1-3**@BLG-gel at pH 7 under sonication for 8 minutes. Before sonication the samples were kept at pH 7 during 36 hours (as in **Figure 7d**) resulting in release of 79-84% of **1-3** prior to sonication.

Comparison of Attempts at PNFs Formation for Samples 1-3@BLG

In all cases when BLG: hydantoin hybrids were heated at acidic pH this resulted in formation of BLGNFs as demonstrated by AFM microscopy (**Figure S8**). **Figure S8a** shows the height image obtained for fibrillated BLG, giving BLGNFs (that is just with protein, without hydantoins, as a reference for comparison), whereas **Figure S8b-d** shows AFM images of BLG fibrillated in the presence of hydantoins **1-2**, resulting in functionalized BLGNFs. In all cases the AFM images show objects with a morphology typical of BLG fibrils with lengths in the micrometer range, consistent with previously reported fibrils from *β*-lactoglobulins. As shown in **Figure S8b-f**, the functionalized fibrils tend to be thicker than normal fibrils (**Figure S8a**).

However, when examined by UV-vis and fluorescence spectroscopy, it was found that during fibrillation (heating at low pH) **1** and **2** underwent dramatic changes in their absorption and fluorescence spectra (**Figure S9-S10**). As often observed, absorbance and fluorescence spectra can undergo large changes upon PNFs formation, therefore control experiments were performed, where aromatic aldehydes (**4** and **5**), used for preparing **1** and **2**, where milled with BLG followed by fibrillation, resulting in the formation of PNFs as demonstrated by AFM (**Figure 2e-f**). Absorption and fluorescence spectra of **4**@BLGNFs and **5**@BLGNFs matched well with spectra obtained from the attempt at forming **1**@BLGNFs and **2**@BLGNFs (**Figure S9-S10**). As **1** and **2** contain a slightly acid labile *N*-acyl hydrazone linker between the fluorescent moiety (generated from an aromatic aldehyde) and the hydantoin scaffold we investigated the behaviour of **3**, more stable towards hydrolysis. **3** was milled with BLG as described above, and the resulting hybrid was dissolved in water and then heated at acidic pH. This resulted in formation of BLGNFs (**3**@BLGNFs). In contrast to **1** and **2**, as shown in Fig. 3 absorption and fluorescence spectra of protein mixed with hydantoin **3** does not significantly change before and after PNF formation (*c.f.* **Figure 2** and **Figure S9-S10**).

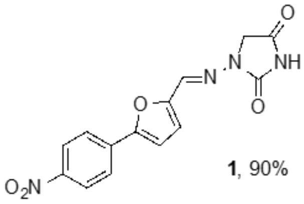

1, 90%

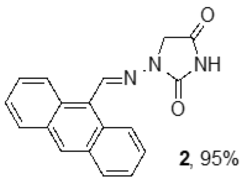

2, 95%

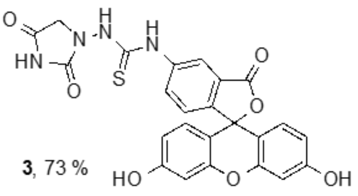

3, 73 %

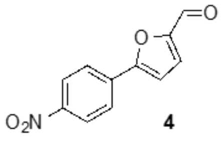

4

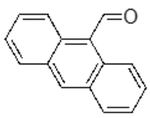

5

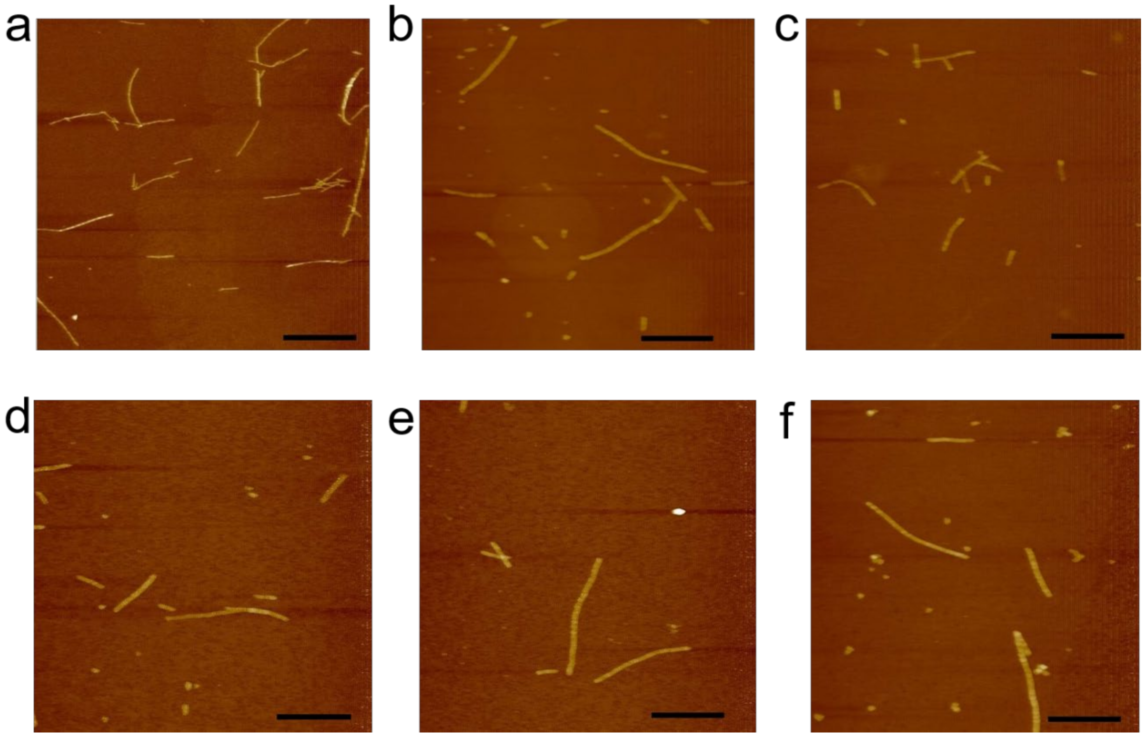

Figure S8. AFM images of BLGNFs formed by heating of BLG or BLG in the presence of **1**-**5**. (a) BLGNFs; (b) Attempt at forming **1**@BLGNFs; (c) Attempt at forming **2**@BLGNFs; (d) **3**@BLGNFs; (e) **4**@BLGNFs; (f) **5**@BLGNFs, scale bars correspond to 1 μm.

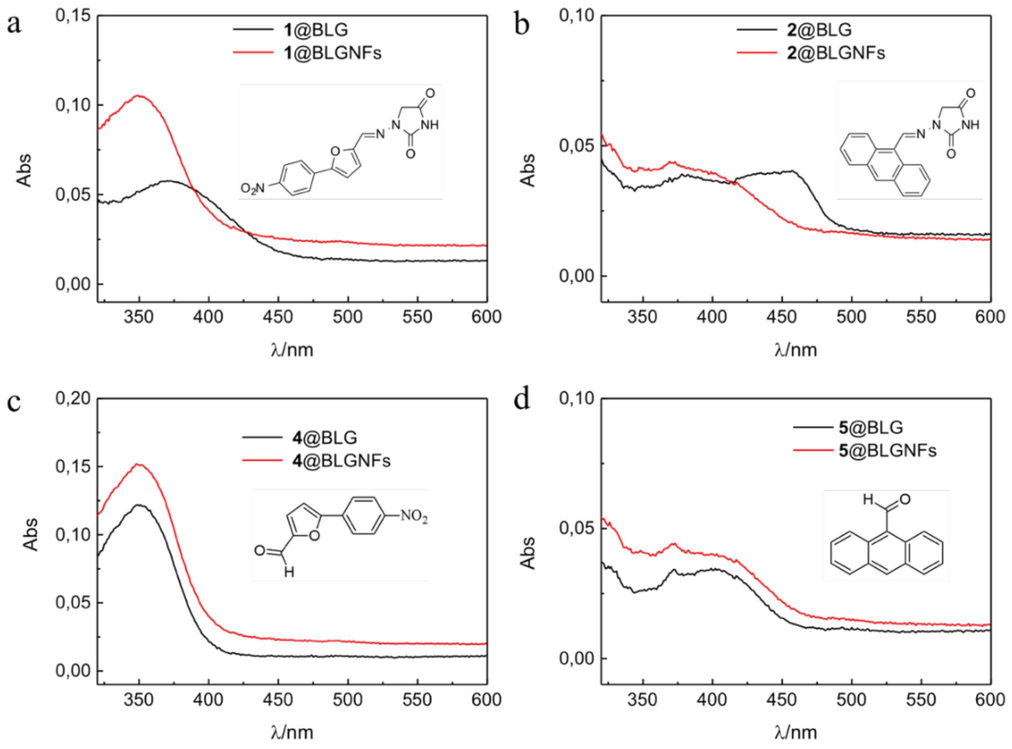

Figure. S9. Absorption spectra of BLG and its complexes. (a): **1**@BLGNFs; (b): **2**@BLGNFs; (c): **4**@BLGNFs; (d): **5**@BLGNFs.

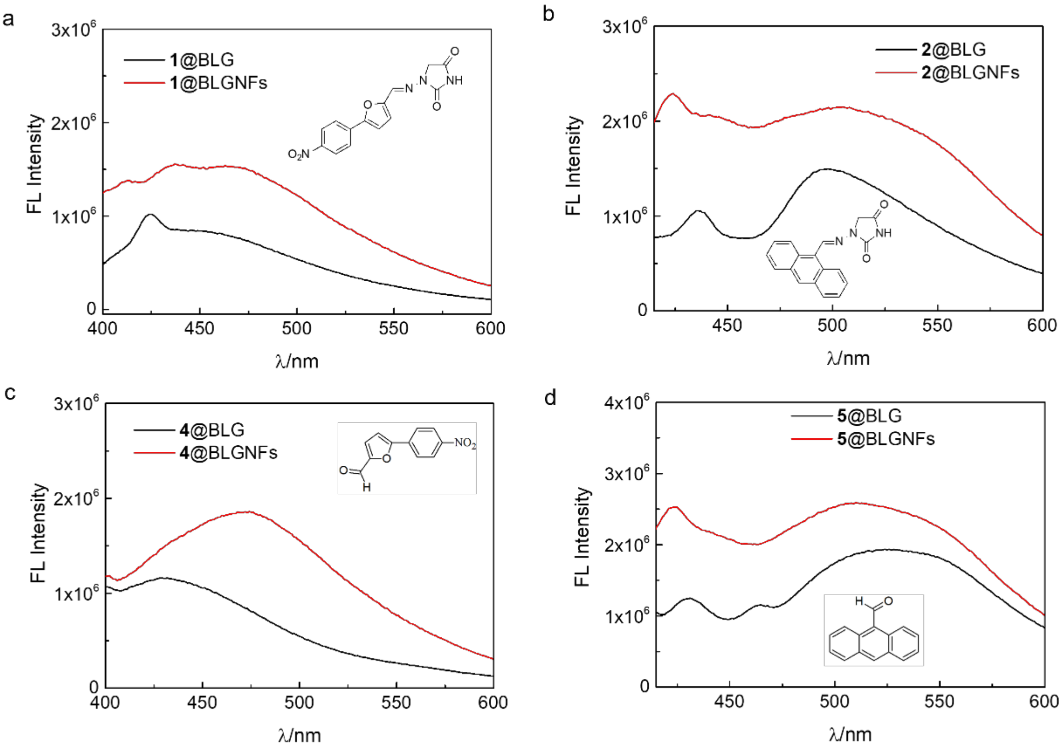

Figure S10. Fluoresecence spectra of BLG and its complexes. (a) **1**@BLGNFs (λ_{ex} = 350 nm); (b) **2**@BLGNFs (λ_{ex} = 370 nm); (c) **4**@BLGNFs (λ_{ex} = 350 nm); (d) **5**@BLGNFs (λ_{ex} = 370 nm).
